# GENE-Counter: A Computational Pipeline for the Analysis of RNA-Seq Data for Gene Expression Differences

**DOI:** 10.1371/journal.pone.0025279

**Published:** 2011-10-06

**Authors:** Jason S. Cumbie, Jeffrey A. Kimbrel, Yanming Di, Daniel W. Schafer, Larry J. Wilhelm, Samuel E. Fox, Christopher M. Sullivan, Aron D. Curzon, James C. Carrington, Todd C. Mockler, Jeff H. Chang

**Affiliations:** 1 Department of Botany and Plant Pathology, Oregon State University, Corvallis, Oregon, United States of America; 2 Molecular and Cellular Biology Program, Oregon State University, Corvallis, Oregon, United States of America; 3 Department of Statistics, Oregon State University, Corvallis, Oregon, United States of America; 4 Center for Genome Research and Biocomputing, Oregon State University, Corvallis, Oregon, United States of America; Oregon State University, United States of America

## Abstract

GENE-counter is a complete Perl-based computational pipeline for analyzing RNA-Sequencing (RNA-Seq) data for differential gene expression. In addition to its use in studying transcriptomes of eukaryotic model organisms, GENE-counter is applicable for prokaryotes and non-model organisms without an available genome reference sequence. For alignments, GENE-counter is configured for CASHX, Bowtie, and BWA, but an end user can use any Sequence Alignment/Map (SAM)-compliant program of preference. To analyze data for differential gene expression, GENE-counter can be run with any one of three statistics packages that are based on variations of the negative binomial distribution. The default method is a new and simple statistical test we developed based on an over-parameterized version of the negative binomial distribution. GENE-counter also includes three different methods for assessing differentially expressed features for enriched gene ontology (GO) terms. Results are transparent and data are systematically stored in a MySQL relational database to facilitate additional analyses as well as quality assessment. We used next generation sequencing to generate a small-scale RNA-Seq dataset derived from the heavily studied defense response of *Arabidopsis thaliana* and used GENE-counter to process the data. Collectively, the support from analysis of microarrays as well as the observed and substantial overlap in results from each of the three statistics packages demonstrates that GENE-counter is well suited for handling the unique characteristics of small sample sizes and high variability in gene counts.

## Introduction

The highly parallelized deep sequencing of cDNA fragments in RNA-Sequencing (RNA-Seq) is the new method of choice in transcriptomics. Its high sensitivity and single-base resolution have contributed substantially to advancing our understanding of gene expression [1]. Recent use of RNA-Seq has led to the identification of a substantial number of new transcripts and their genes, an appreciation into the abundance of a diversity of transcript isoforms as well as the diversity of alternative transcriptional start sites [2]–[5]. RNA-Seq has also been applied to areas of transcriptomics that in the past, were difficult to study, such as RNA editing, allele-specific expression, and study of expression changes in single cells as well as co-cultivated organisms [6]–[8].

RNA-Seq can be used to quantify and study genome-wide changes in gene expression. Such applications typically start with aligning RNA-Seq reads to a reference sequence to identify all expressed genome features. The numbers of reads per feature are then calculated to derive feature counts and infer expression levels. Finally, a statistical test is applied to normalized feature counts, followed by a collective assessment of significance based on an acceptable false discovery rate (FDR), to identify differentially expressed features with statistical significance [9]. From this point on, we will simply refer to features as genes.

While the use of RNA-Seq for quantifying gene expression is relatively straightforward to conceptualize, RNA-Seq experiments have considerable computational and statistical challenges. The massive quantities of short reads require ultra fast alignment programs that adequately address memory demands. The volume of data is also of concern if the end user desires systematic storage and management, as well as integration of data into third party software for additional analyses. Importantly, the combination of a large number of comparisons and small sample sizes causes more concern than usual about the power of the statistical test.

The small sample sizes rule out the uncritical use of methods that rely on large-sample asymptotic theory. Elementary tools for the Poisson distribution will over-state differential expression because of overdispersion, the phenomenon where the count variability between biological replicates is substantially greater than that predicted from the Poisson model [10]–[12]. Failure to address overdispersion will cause the model to incorrectly interpret large variation between biological replicates as evidence of differential expression and provide drastically misleading conclusions [13].

The negative binomial (NB) distribution offers a more realistic model for RNA-Seq count variation and still permits an exact (non-asymptotic) test for differential gene expression [11], [14]. For each individual gene, a NB distribution uses a dispersion parameter to model the extra-Poisson variation between biological replicates. When considering all genes in an RNA-Seq experiment, statistical power of the exact NB test can be gained by sensibly combining information across genes to estimate the dispersion parameter. The constant dispersion version of the edgeR package, for example, estimates a single dispersion parameter for all genes [11], [14].

The assumption that a single parameter is constant across all genes is, however, not met for RNA-Seq data [13]. To address this, the edgeR package (version 2.0.3) includes an option for empirical Bayes estimation of the dispersion parameter for each gene, with shrinkage towards a common value as well as a ‘trend’ option that shrinks towards a value determined by nonparametric regression of the dispersion parameter on the mean [15]. The DESeq package, also based on the NB distribution, employs nonparametric regression to estimate the dispersion parameter as a function of the mean and treats the estimated dispersion parameters from this model as known [10]. The NBPSeq package uses a test based on a simple over-parameterized version of the NB distribution called the NBP where an additional parameter is introduced to allow the dispersion parameter to depend on the mean [13].

Some computational pipelines such as Cufflinks, Myrna, and ArrayExpressHTS have been developed for analysis of RNA-Seq data for expression changes [12], [16], [17]. Cufflinks is a pioneering pipeline that combines RNA-Seq alignment with inference of transcript isoforms directly from the RNA-Seq reads, and assessment of differential expression of the inferred transcripts [16]. Cufflinks has been updated to use a test based on the NB distribution (http://cufflinks.cbcb.umd.edu/). Myrna can use cloud computing to cost-effectively exploit large computational resources. With this pipeline, only permutation and large-sample likelihood-ratio tests were considered, which do not sufficiently address small sample sizes or the mean-variance dependence in RNA-Seq data [12], [13]. ArrayExpressHTS is an R/bioconductor-based pipeline that combines processing, data quality assessment, a variety of alignment programs, inference of transcript isoforms, and statistical analysis with Cufflinks or MMSEQ [18]. The latter provides an estimate of expression levels but does not identify differentially expressed genes.

We describe GENE-counter, a simple pipeline with the appropriate statistical tests for studying genome-wide changes in gene expression. GENE-counter is modular and flexible to allow the end user to use different alignment programs, easily change parameters, and use different statistical tests for analysis of differential gene expression and enriched gene ontology (GO) terms. Results are transparent and systematically stored in a MySQL database, a standard format usable by most third party software. To test GENE-counter, we developed a pilot RNA-Seq dataset from *Arabidopsis thaliana* elicited for PAMP-triggered immunity (PTI). In PTI, recognition of conserved pathogen-associated molecular patterns (PAMPs) leads to a number of induced responses, including genome-wide changes in expression that can be detected 6∼7 hours post inoculation (hpi) [19]. PTI is intensively studied and has a correspondingly extensive resource of publicly available microarray data that we used for comparative purposes to support our findings. RNA-Seq data were analyzed using GENE-counter and results were well supported by other statistics packages as well as analysis of microarrays. We also compared the performance of GENE-counter to Cufflinks and showed that with these data, results from the two pipelines were considerably different.

## Materials and Methods

### Design and implementation of GENE-counter

We used a combination of Perl, MySQL, R, as well as C++ software (CASHX) to develop GENE-counter. Perl handles the decision logic for the overall pipeline flow to call different software packages for specialized needs, such as data storage and querying, statistical analysis, and fast short-read alignment, which were developed using MySQL, R, and C++, respectively. Perl is also used to handle the user-interface implementation of GENE-counter.

GENE-counter has five tools:

#### Configuration tool

This tool is used to configure GENE-counter to leverage available resources, minimize computational overhead, and reduce duplication of effort. There is potential for multiple users to connect to the same reference sequence database with one or more read databases. Similarly, an end user has the option to align the sequences from their read database to multiple installed reference sequence databases, such as different versions of the same genome sequence. All subsequent gene count and alignment data will be stored in an alignment database for each end user. This flexibility enables easy switching of read databases and/or alignment databases to test and compare results produced by GENE-counter when used with different settings such as alignment parameters.

#### Processing tool

This tool includes two modules for processing RNA-Seq reads and aligning sequences to a reference sequence, respectively. In the first module, user-defined information is recorded to describe the RNA-Seq experiment, such as treatments, replicate numbers, date, etc. The RNA-Seq reads are processed to identify and enumerate the occurrences of each unique sequence within each replicate. Unique RNA-Seq sequences, their occurrence, and an assigned identification number populate the read database. GENE-counter can use RNA-Seq reads produced from any of the next generation sequencing (next-gen) platforms but limited information is stored if a platform other than Illumina is used.

The second module aligns all unique RNA-Seq sequences to features of a reference sequence database. Any alignment program that can output alignments in the SAM format can be used [20]. We configured GENE-counter with CASHX version 2.3, Bowtie, and BWA [21], [22]. CASHX version 2.3 is the default alignment tool. End users will need to configure other alignment programs if desired.

GENE-counter, by default, will generate gene counts using the best alignments produced with the desired alignment program settings, which are easily set by the end user. For instance, if set to allow a maximum of two mismatches, GENE-counter first relies on alignments with perfect matches, after which it will also use alignments that had one and then two mismatches that did not produce alignments with fewer mismatches. The alignments, in conjunction with their read occurrences, are used to derive gene counts for each reference sequence feature. Data are systemically stored in the alignment database.

#### Assessment tool

This tool can be used to assess the quality of the data. The assessment tool interrogates the alignment database and produces summary files that display raw count data, summary counts for types of features annotated in the reference sequence, and intraclass correlation coefficient (ICC) values for replicates. The ICC is a descriptive statistic that can be used to quantify the degree of resemblance of quantified measurements of samples within a defined group. To derive ICC values, counts are normalized to reads per quarter million after incrementing by one to handle zeroes prior to log transformation and the ‘irr’ package in R is used to calculate ICC using the log transformed counts [23], [24]. There is no absolute ICC value that determines useable versus unusable replicates. Rather, the end user can inspect the values as a gauge of the quality of the replicates.

#### Statistics tool

This tool uses the NBPSeq statistics package as the default method for assessing the normalized gene counts to produce a list of differentially expressed genes [13]. GENE-counter is also configured for the edgeR and DESeq statistics packages [10], [15]. Normalization was implemented using the built-in normalization methods of each statistics package. For NBPSeq, the function nbp.test() is called with the appropriate counts and parameters, and normalization occurs automatically followed by differential expression analysis. For edgeR, the ‘estimateTagwiseDispersion()’ function was used, with the ‘trend’ parameter set to true and using the matrix counts produced by the ‘estimateCommonDisp()’ function, to read in the matrix of read counts and normalize counts as well as estimate the dispersion parameters [15]. The ‘exactTest()’ function was used to calculate p-values for each gene. For DESeq, the ‘newCountDataSet()’ function was used to generate a cds object from the matrix of read counts and a subsequent call to the ‘estimateVarianceFunctions()’ was used to generate the variance estimates [10]. The ‘nbinomTest()’ function was called to generate the p-values for differential expression.

The conclusion about evidence for differentially expressed genes is subsequently based on an ordering of p-values and a cutoff for statistical significance to adhere to acceptable false discovery rates [9]. The ‘qvalue’ package in R was used to generate q-values using the p-values generated by the respective statistics packages.

#### GORich tool

The list of differentially expressed genes can be analyzed for enriched gene ontology (GO) terms using any one of three tests available: the parent-child-inheritance, term-for-term, and GOperm analysis methods [25]–[27].

#### Data storage

GENE-counter records reference sequence definitions, RNA-Seq read sequence alignments, and derived gene count data, in a MySQL relational database.

Details in installing and using GENE-counter are provided in the user's manuals.

### Improvements to CASHX

A number of changes were made to CASHX version 1.3 [28]. We implemented a simple hashing algorithm that eliminated empty containers corresponding to preamble sequences absent from reference sequences. We further compressed the database to only store corresponding reference sequence coordinates for each of the indexed k-mers. We also changed the order in which information was stored within each container. The reference sequence coordinates for each k-mer within a preamble container are now sorted based on the sequence of the 16 nucleotides following the preamble, allowing for sorting of 64 bit integers (2 bits for each nucleotide). Implementation of a simple binary search algorithm dramatically reduced the search time within a preamble container by an order of magnitude. Finally, we implemented a mirrored search logic to index reads to their corresponding container(s), similar to the method employed by Bowtie [21]. Two equal-length fragments derived from each query read are used to seed alignments of the read. CASHX uses the integer converted from the seed fragments and increments their integers through all possible mismatch combinations.

Mapping programs were benchmarked in a single thread on a CentOS 5.1 8 Intel Xeon X5355 x86 64-bit processor with 2.66 GHz and 32 GB RAM. For Bowtie and SOAP2, version 0.12.3 and 2.20, respectively were used [21], [29].

### Developing the *Arabidopsis thaliana* reference database

We developed a comprehensive reference database using the genome and transcript annotations in the TAIR9 genome release (www.arabidopsis.org/). The Generic Feature Format (GFF3) file was used to populate a MySQL database with information such as genes, their classifications (e.g. coding, transposable elements, pseudogene, etc.), transcript classifications (mRNA, miRNA, tRNA, rRNA, etc), coordinates, gene features, and the corresponding gene isoforms. Also included were over 18,000 sequences corresponding to splice junction sequences [3].

Information on how GENE-counter can be used to derive count data from either a list of gene features in a reference genome, or transcript features in a reference transcriptome can be found in GENE-counter's user's manual.

### RNA preparation and sequencing

Bacteria were grown in King's B media and infiltrated into plants as previously described [30]. Briefly, we used a syringe lacking a needle to infiltrate the abaxial side of leaves of six-week old Arabidopsis plants. Plants were infected with either the Δ*hrcC* mutant of *Pseudomonas syringae* pv. *tomato* DC3000 (*Pto*DC3000) or mock inoculated with 10 mM MgCl_2_ 7 hpi. Each treatment was done as biological triplicates with each pair of replicates done at separate times and derived from independently grown plants and bacteria. Total RNA was extracted from leaves at 7 hpi, enriched for mRNA using Poly(A)Purist (Ambion Inc., Austin, TX) and processed for RNA-Seq as described [31]. The replicates were sequenced one per channel using the 36-cycle sequencing kit on an Illumina. Sequencing was done by the Center for Genome Research and Biocomputing core facility at Oregon State University (CGRB; OSU).

### Pre-processing and aligning RNA-Seq reads

Prior to processing, the first six and last five nucleotides from each RNA-Seq read were trimmed. Reads were then aligned allowing up to two mismatches in the alignment as specified in the global configuration file found in GENE-counter; this setting can be changed by the end-user. Only RNA-Seq reads that aligned to features of a single gene locus were considered, which we referred to as unambiguous and useable reads. In cases where a read sequence aligns to a single gene locus but to multiple gene isoforms, GENE-counter assigned the reads equally to each of the mapped isoforms. Furthermore, to be considered for differential expression, genome features were required to have assignments in all replicates of at least one of the treatments. Settings can be easily modified at the command line when running the statistics tool of GENE-counter.

GENE-counter was benchmarked in a single thread on a CentOS 5.1 8 Intel Xeon X5355 x86 64-bit processor with 2.66 GHz and 32 GB RAM.

### Derivation of MA plot

M was calculated as the difference between the log_2_ average of GENE-counter normalized values for all replicates in Δ*hrcC* and MgCl_2_ (log_2_(Δ*hrcC*)−log_2_(MgCl_2_)). A was calculated as the average of all log_2_ transformed GENE-counter normalized counts (1/2 * ((log_2_(Δ*hrcC*)+log_2_(MgCl_2_))). All normalized counts had 1 added to them prior to log transformation to avoid problems with zeroes.

### Comparing results from GENE-counter with different statistics packages

Gene expression was calculated by natural log transformation of the average number of raw gene counts for all genes. The percentage of genes was plotted per expression quantile. The plot was generated using the ‘plot’ function in R [24]. All genes were also ranked according to the p-value assigned by the respective statistics package and used to create a scatter plot of all genes found significant in pair wise comparisons. Linear regression lines were plotted using the ‘lm’ function in R [24].

### Analysis of NBPSeq normalization

The findDGE.pl script of GENE-counter was run 1000 times to examine the effects of random thinning used by NBPSeq to normalize gene counts. For each iteration, a random seed was supplied to the ‘-s’ option of the findDGE.pl script to randomize the thinning process. The percentage of times each gene from the original NBPSeq set of 308 induced genes was determined and plotted against their original q-values. The q-value bins were categorized in quantile increments of 0.005.

### Analysis of microarrays

The mRNA labeling, hybridization, and scanning of Affymetrix ATH1 microarrays were done by the CGRB core facility at OSU. Microarrays were normalized using RMA [32]. Significance was determined based on the overlap of genes common to each of four methods: BRAT (corrected p-value≤0.3) [27], LIMMA (p-value≤0.1) [33], [34], PaGE (confidence level ≥0.85) [35], and SAM (q-value %≤10%) [36].

To compare against results from analysis of RNA-Seq, a log_2_ scatter plot was produced. For the RNA-Seq data, the fold-change values were calculated using the GENE-counter normalized values (Δ*hrcC* versus MgCl_2_). For the Affymetrix ATH1 data the raw fluorescence values were used to calculate the normalized fold-change values using the Robust Multi-array Analysis normalization method [37]. The log_2_ values were calculated for both ratios, and the RNA-Seq data (y-axis) was plotted against the Affymetrix ATH1 data (x-axis). Estimated regression lines and Pearson's correlation coefficient were calculated using the ‘lm()’ function and the ‘cor()’ functions in the R programming language, respectively [24].

### Cufflinks

The same set of unambiguous and usable reads from each replicate used by GENE-counter, were also used for analysis by Cufflinks. Reads were mapped to the genome reference sequence using either Tophat version 1.1.2. with the flags ‘–library-type fr-unstranded -m 2’ or Bowtie with the flags ‘-v 2 -f -a –best –strata –S’ to most closely match alignment parameters used in running GENE-counter (allowing for up to two mismatches and choosing the best alignments). Bowtie alignments were converted to BAM and sorted for use with Cufflinks using SAMtools version 0.1.6 [20]. Cufflinks version 1.0.2 was run using default parameters on each replicate file. Each replicate ‘transcripts.gtf’ file created by Cufflinks was then merged with the Arabidopsis annotation using Cuffmerge with the final merged annotation file being used in Cuffdiff as the reference genome annotation. Cuffdiff version 1.0.2 was run to most closely emulate the way GENE-counter data was used by throwing the flags ‘–emit-count-tables -c 1 –FDR 0.05’ with the ‘-b’ flag being supplied the Arabidopsis reference genome sequence in order to use bias correction.

## Results and Discussion

**Figure 1:**
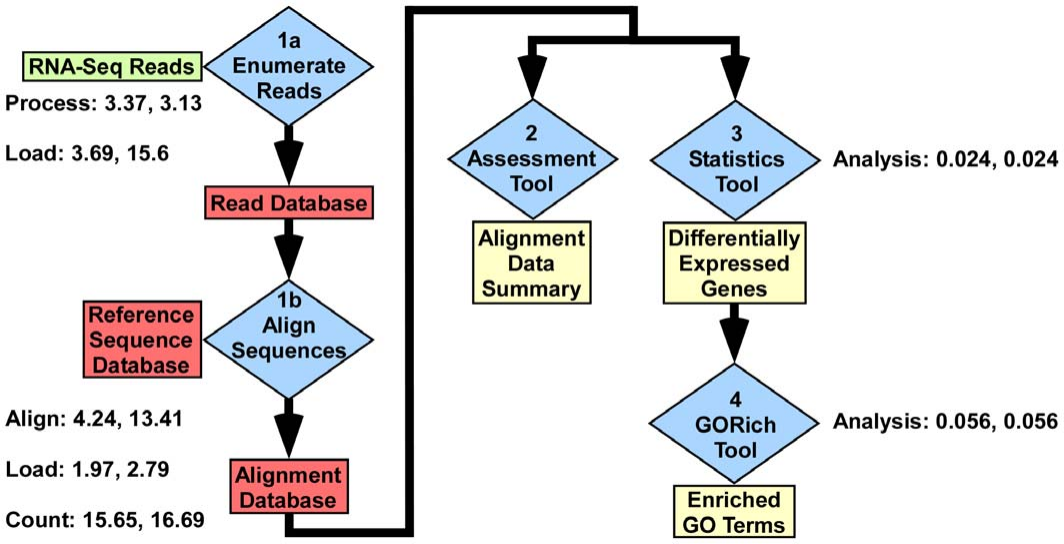
Entity-relationship diagram for four tools of GENE-counter. Each tool is numbered indicating the order in which data is typically processed: **1a and 1b**) the two modules of the processing tool, **2**) the assessment tool, **3**) the statistics tool, and **4**) the GORich tool. The processing tool uses a directory of FASTA files for each replicate as an input (RNA-Seq reads) to tabulate a list of unique read sequences and enumerate the occurrence of each read sequence within each FASTA file. Data are stored in a read database. The processing tool uses a SAM compliant alignment program to align and assign read sequences to features stored in a user-developed reference sequence database. Alignment information and associated count data are stored in the alignment database. Results can be analyzed by the assessment tool to produce an alignment summary, which includes a summary report of replicates and intraclass correlation coefficient (ICC) values. For statistical analysis, the statistics tool can use the NBPSeq, trend version of edgeR, or DESeq statistics package to assess the normalized gene count data. Results are produced as a list of differentially expressed genes, their associated gene counts, normalized gene counts, p- and q-values. The GORich tool can be used to identify enriched Gene Ontology (GO) terms in a list of differentially expressed genes. Three different methods are provided. The amount of time (hours) for steps to analyze over half a billion RNA-Seq reads is shown (GENE-counter running eight instances of CASHX with no throttle control and one instance of Bowtie with maximum throttle control (separated by a comma).

We developed GENE-counter as a modular pipeline with five tools for processing, aligning, analyzing, and storing RNA-Seq data (Fig. 1; see material and methods). Perl is used to handle the user-interface of GENE-counter, which makes its use relatively easy by only requiring the end user to be familiar with simple commands at the command line.

GENE-counter stores all processed data in a standard relational database and each of its tools therefore use the standard structure query language (SQL) to retrieve data. Thus, in order to run GENE-counter, it requires configured read, alignment, and reference sequence databases. The first two databases will be populated while running GENE-counter to contain the RNA-Seq reads and alignment information, respectively. The reference sequence database should be populated with reference sequences as well as annotation information prior to running GENE-counter. The three databases will be interrogated by each of the tools of GENE-counter to manage and analyze the data.

### Processing tool: alignment programs

The modularity of GENE-counter gives end users a preference in configuring any SAM compliant alignment program. The default configured option is an improved version of the CASHX alignment program [28]. The improved CASHX, version 2.3, is SAM compliant, and like its predecessor, uses a 2 bit-per-base binary format to compress both the RNA-Seq reads and reference sequence database to exhaustively find all possible alignments that meet user-specified criteria [20]. The improvements to CASHX allowed for mismatch alignments and dramatically increase alignment speed to reduce the time for aligning sequences by almost 20× and memory demands by 1.5× without compromising accuracy (Table 1).

**Table 1:** Benchmarking CASHX ver. 2.3.

Mapping program*	Clock time (min)	Peak memory usage (Mb)	Alignments identified	Missed alignments (% of the ∼8.8 million expected found)†	Unsupported alignments‡
**0 mismatches** §					
CASHX ver. 2.3	3.70	2.32	8,815,743	0 (100%)	0
CASHX ver. 1.3	73.23	3.48	8,815,743	0 (100%)	0
SOAP2	1.71	0.79	8,815,745	2 (<100%)	4
Bowtie	3.22	0.13	8,815,743	0 (100%)	0
**2 mismatches** §					
CASHX ver. 2.3	16.32	2.32	9,138,971	0 (100%)	0
SOAP2	8.85	0.81	9,094,436	44,576 (100%)	41
Bowtie	20.38	0.19	9,138,971	0 (100%)	0

*CASHX ver. 1.3 does not allow for mismatches and was not benchmarked for all tests [21], [28], [29].

§ We derived a simulated RNA-Seq dataset from 8,815,743 regions of the Arabidopsis genome that were unique in sequence and lacked any Ns for use in benchmarking CASHX ver. 2.3.

† For no mismatches, values are based on expected unique alignments; for two mismatches, values are based on the number of alignments confirmed by at least two software programs.

‡ Number of alignments that were not confirmed by at least one of the other tested software programs.

We benchmarked the CASHX ver. 2.3 alignment program against Bowtie and SOAP2 that, like several alignment programs, use the Burrows Wheeler Transformation compressed index to reduce computational weight and increase speed [21], [29] (Table 1). Using simulated data, in which we knew the exact alignments, CASHX and Bowtie were identical in accuracy but slower than SOAP2 in regards to speed. CASHX was marginally faster than Bowtie when mismatches were allowed and showed a greater advantage in alignment time as the size of the dataset increased (data not shown). In contrast, CASHX had a fairly substantial memory demand relative to the other two tested alignment programs. Though, as the number of reads increased, memory demands by SOAP2 exceeded that of CASHX (data not shown).

The memory demands are potentially limiting or end users may simply be less familiar with CASHX. To address these possibilities, we configured GENE-counter for two other alignment programs, Bowtie and Burrows-Wheeler Alignment tool (BWA) [21]. Other options to control memory demands include running fewer instances of alignment programs or using the built-in throttling mechanism to specify the number of sequences processed at a time. We did not exhaustively benchmark BWA or any other alignment programs in the same manner as presented in table 1. We therefore recommend end users to test their alignment program of preference prior to use with GENE-counter. Nonetheless, when the accuracy of alignment by BWA was examined using reads from a pilot RNA-Seq experiment (see below), results suggested that BWA was similar to CASHX and Bowtie. We did observe differences in how each of the three programs aligned reads with ambiguous bases and used best alignments (data not shown). The default of CASHX is to exclude reads with ambiguous bases and use only the best alignment.

### Benchmarking GENE-counter

We processed 522 million RNA-Seq reads of 40 nt in length to demonstrate extremes in running parameters of GENE-counter (S. A. Filichkin and T. C. Mockler, unpublished). In one, we maximized speed at the expense of memory by using eight instances of CASHX in the absence of throttle control. The entire process took GENE-counter ∼29 hours and memory demands peaked at 17 GB to analyze the greater than half billion RNA-Seq reads (Fig. 1). Similar running parameters using BWA took ∼30 hours and memory peaked at 5 GB [22]. In another setting, we emphasized memory demands over speed by using only one instance of Bowtie and maximum throttling to limit memory usage [21]. GENE-counter took ∼52 hours but memory demands peaked at only ∼1 GB. In both cases, up to two mismatches were allowed and all steps, from populating the read database with raw RNA-Seq reads to assessing data for enriched GO terms, were measured. These examples demonstrate the range in versatility and scalability of GENE-counter to flex to the size of the RNA-Seq experiment and operate within the limits of an end-user's computer hardware. Running times will vary depending on hardware.

Storing and interrogating information in databases adds a considerable amount of analysis time by GENE-counter. Although this could be considered a disadvantage, it is offset by the substantial timesaving that will be gained in downstream analyses. Most production level desktop and web-based software platforms have application program interfaces (APIs) that interact with MySQL. These data can therefore be easily queried using third party programs. For example, alignment data processed by GENE-counter can be easily pulled into the generic Genome Browser (GBrowse), a robust web-based platform for visualizing genomes, gene features, and expression data [38]. The systematic storage of data contributes to the modularity of GENE-counter and gives each of the tools a high degree of independence, which allowed for the easier path in configuring different alignment programs and statistics packages. It also gives software developers the ability to leverage the comprehensive data querying language of MySQL to quickly extend the utility of GENE-counter to accelerate development of additional analytical methods and distribution tools. If time is of concern, end users can use a preferred alignment program to derive gene counts independent of GENE-counter and provide counts directly to the statistics tool. However, alignment data will not be stored.

### Analysis of a pilot RNA-Seq dataset

To examine the efficacy of the entire GENE-counter pipeline, particularly the analysis of differential gene expression, we developed a small-scale RNA-Seq dataset using the intensively studied defense response of Arabidopsis (E-GEOD-25818; http://www.ebi.ac.uk/arrayexpress/). We chose this response because of the availability of microarray data that we could use to support results. We isolated, prepared and sequenced cDNA preparations derived from biological triplicates from Arabidopsis infected with either a Δ*hrcC* strain of *Pto*DC3000 or mock inoculated with 10 mM MgCl_2_ 7 hpi. The Δ*hrcC* strain has a mutation that affects the assembly of the type III secretion system (T3SS). The T3SS is an apparatus required to inject type III effector proteins, which collectively dampen host defenses, directly into plant cells [39], [40]. Without the T3SS, strains are nonpathogenic and elicit PTI.

GENE-counter took ∼3.0 hours when eight instances of CASHX were run in parallel, to process and analyze the ∼54 million 25 nt-long reads. For the alignments, we allowed up to two mismatches. On average, ∼63% of the reads from the Δ*hrcC*-challenged and mock-inoculated Arabidopsis RNA-Seq experiment aligned to the reference sequence database. We further required GENE-counter to only consider reads that aligned to a single annotated feature of an expressed gene, such as 5′ and 3′ UTRs, exons, splice junctions, and retained introns. Approximately 50% of the total reads met this additional criterion and were termed unambiguous and usable. Thus, based on the replicate with the fewest number of unambiguous and usable reads and our requirement for a feature to be aligned with reads in all replicates of at least one treatment, 20,045 of the 33,518 genes annotated for Arabidopsis were considered expressed. Intraclass correlation coefficient (ICC) values for the Δ*hrcC* and mock treatments were both considered acceptable with values of 0.8 and 0.88, respectively [23]. The ICC is a quantitative statistic for assessing the degree of similarity of values within a group.

### Statistics tool

The trend version of edgeR, as well as the DESeq and NBPSeq statistics packages use different ways to model the NB dispersion parameter as a function of the mean [10], [15]. The three are similar in the exact test they use and each method provides the same power benefit associated with combining information across genes [13]. We demonstrated through systematic simulation studies that in terms of statistical power and control of false discoveries, the three methods performed similarly to each other and substantially better than alternative test procedures such as *t*-test, a test based on Poisson model, and the constant or moderated dispersion versions of edgeR [13]. We therefore configured GENE-counter with each of the three statistics packages. Since Perl handles the user-interface, end users are not required to use the R statistics programming language.

The NBPSeq package was implemented as the default method and represents the first known practical use of the NBP distribution. The NBP model has the advantage of relative transparency and model simplicity. The NBP does not require the input of any user-defined parameters. In contrast, tuning parameters are employed by the trend version of edgeR and DESeq to control smoothing of mean-variance and mean-dispersion curves [10], [15]. How to find the best tuning parameters is still a topic of research. Additionally, while these two other methods provide more flexibility, they also run the risk of overfitting and are prone to the impact of potential unstable variance estimation in the extreme range of expression levels, or ‘boundary effects’ [13].

**Figure 2:**
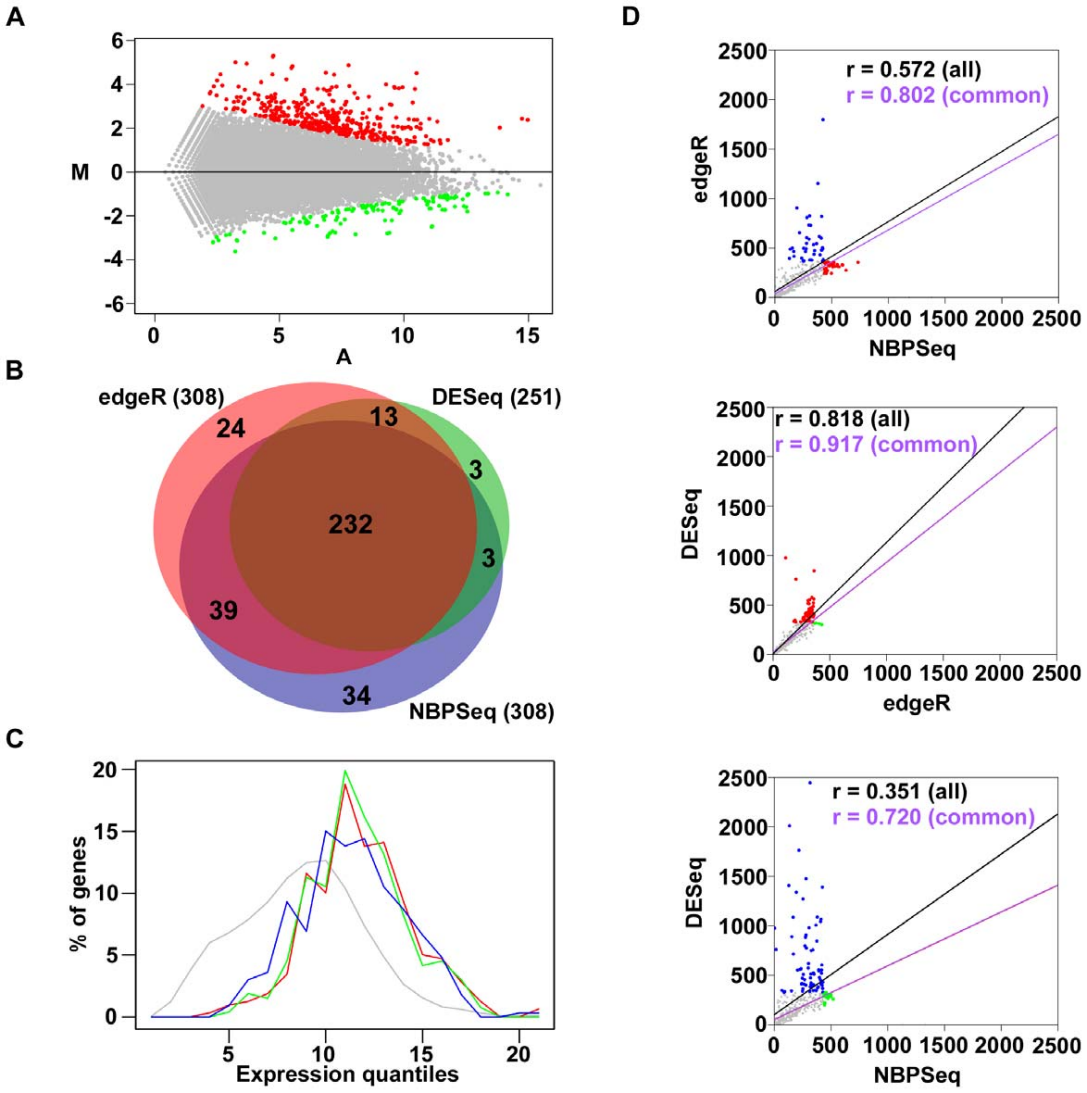
Analysis of RNA-Seq data for genes differentially expressed in Arabidopsis infected with Δ*hrcC* relative to mock inoculation 7 hpi. (**A**) The differentially expressed genes identified between Δ*hrcC*- and mock-treated Arabidopsis. Results are plotted using an MA-based method. Differentially expressed genes were identified using GENE-counter with the NBPSeq statistics package. Induced and repressed genes are highlighted in red and green, respectively (FDR≤5%). (**B**) Area-proportional Venn diagram comparing the differentially induced genes identified using GENE-counter running NBPSeq, the trend version of edgeR, or DESeq. Read counts were normalized using the methods provided in each statistical package prior to analysis (FDR≤5%). (**C**) Distribution of gene expression levels. Percentages of total genes (y-axis) were categorized per expression quantile, increasing from left to right (x-axis; natural log transformation of average number of normalized aligned reads per gene): gray; all genes; blue, red, and green; differentially induced as identified using GENE-counter running edgeR, DESeq, or NBPSeq, respectively. (**D**) Pair-wise comparisons of p-value rankings for genes identified as significant. Genes were color-coded gray if identified by both statistical packages, blue, red, or green, if uniquely identified by GENE-counter running NBPSeq, edgeR, or DESeq, respectively. Regression lines are plotted based on all genes (black) or only those common to both statistical packages (red). Pearson's r values are shown and colored accordingly.

With a FDR≤5%, GENE-counter running NBPSeq, returned a list of 308 differentially induced and 79 repressed genes in Δ*hrcC*-infected plants relative to mock-inoculated plants (Fig. 2A; Table S1; from hereafter referred to as the ‘original NBPSeq set’). GENE-counter running the trend version of edgeR and DESeq identified 308 and 251 induced genes, respectively (Fig. 2B). Of these, 88% and 94% of the genes, respectively, were also in the original NBPSeq set. We plotted the genes identified from the three methods on an expression scale to examine the effects of gene expression levels on detection of differential expression (Fig. 2C). In general, the three methods captured broad and very similar distributions of gene expression levels. A fair proportion of genes unique to edgeR and DESeq were concentrated in the middle of the expression scale, giving a pronounced sharp peak where results from NBPSeq showed more of a plateau. The genes uniquely identified were found distributed throughout the expression scale.

We also compared the p-value rankings for the induced genes identified from each statistical package (Fig. 2D). Again, in general, there were good correlations in rankings between all pair wise comparisons. For the genes uniquely identified by one method but not the other, the unique genes were still nevertheless highly ranked, typically within the top ∼2.5% or 500 of the ∼20,000 ranked genes. Our results confirmed our previous findings that all three statistics packages were comparable and therefore suitable options in GENE-counter [13].

In order to use an exact NB test, which does not rely on large-sample asymptotics for assessing differential gene expression, the three statistics packages need to normalize the counts. In other words, the total numbers of reads must be approximately equal in all replicates. The edgeR method uses quantile adjustment, DESeq adjusts the counts by scaling and NBPSeq adjusts gene counts by random thinning [10], [15]. Normalization is suggested to potentially affect the sensitivity of RNA-Seq analysis [41]. With the data tested here, similar results were produced from GENE-counter when run with each of the three different statistics packages, including their corresponding methods for normalization. This observation suggested that the different normalization methods did not have large effects on the results (Fig. 2).

**Figure 3:**
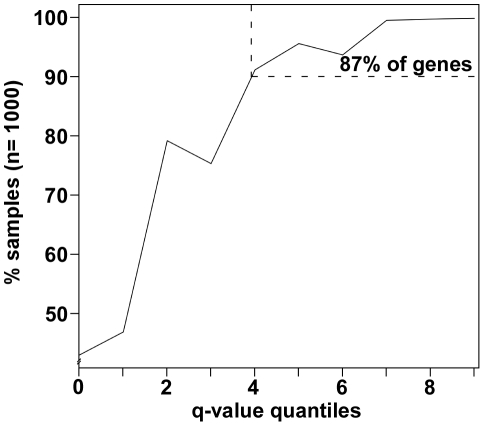
Analysis of NBPSeq normalization on differential expression. The percent of iterations a gene from the original set was identified as differentially induced (y-axis; n = 1000) was plotted as a function of q-value (x-axis; q-values determined for the original set of differentially induced genes categorized in quantile increments of 0.005 from least significant (q-value = 0.05) on the left to most significant (q-value = 0) on the right). For each iteration, different random number seeds were used to randomly thin gene counts. The percentage of genes found in ≥90% of the samples is indicated.

The adjusting of gene counts by random thinning will yield slightly different normalized counts by separate analyses. This method, however, does not have substantial consequences to the overall conclusions on differential gene expression. As evidence, we analyzed results from running GENE-counter 1000 times with NBPSeq and randomly thinned gene counts (Fig. 3). As expected, the trend in consistency of differential expression correlated strongly with increasing significance of q-values. Of the original NBPSeq set of 308 differentially induced genes, 87% were identified as differentially induced in ≥90% of the samples (Fig. 3). Thus, in general, the great majority of genes were consistently identified and thinning will not have substantial impacts on conclusions. There are however, some instances where random thinning could be viewed as undesirable, e.g., one replicate is severely under-sequenced relative to all others. We would encourage an end user to re-sequence the replicate. Nevertheless, an alternative option would be to use one of the other configured statistics packages of GENE-counter.

### Analysis of enriched GO terms

A careful inspection of descriptions of the original NBPSeq set of differentially induced genes found that 36% of the annotated genes functions were in plant defense or were identified based on differential expression in response to pathogens, wounding, and/or stresses. Another 15% were annotated as being involved in signal perception, transduction, secretion or modification of the plant cell wall. We also analyzed the induced genes using the parent-child-inheritance method available in the GORich tool of GENE-counter and found 124 enriched GO terms (Table S2) [26]. We compared these to enriched GO terms of genes identified from publicly available microarray studies of plant defense [42]–[49]. A total of 88 enriched GO terms associated with the differentially induced genes were found associated with at least one other microarray study; 62 were found in at least three of the studies. We concluded that the original NBPSeq set of differentially induced genes was similar to those previously found using analysis of microarrays.

### Comparisons with analysis of microarrays

We used analysis of microarrays as an alternative technical method to globally assess differential induction and provide independent support for the original NBPSeq set of induced genes. We hybridized the same mRNA samples to Affymetrix ATH1 microarrays and identified 366 induced genes (Table S3; GSE25818; http://www.ncbi.nlm.nih.gov/geo/). For comparisons between RNA-Seq- and microarray-based expression studies, we limited the analysis to only genes that were detectable by both methods. As a result, 254 (82%) and 364 (99%) of the genes identified using GENE-counter or analysis of microarrays, respectively, could be compared.

**Figure 4:**
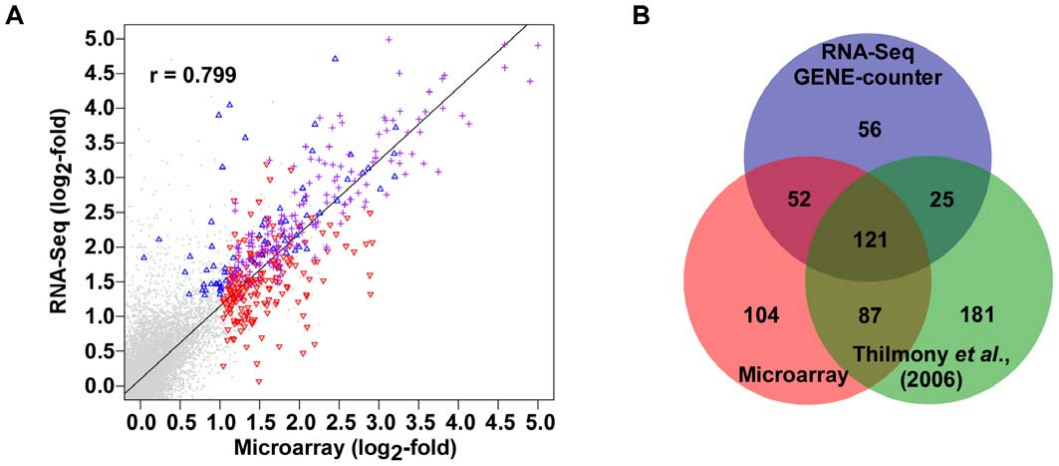
Comparison of analysis of RNA-Seq with analysis of microarrays. (**A**) Comparison of estimated log_2_-fold changes from analysis of microarrays (x-axis) and RNA-Seq using GENE-counter running NBPSeq (y-axis). Only induced genes measurable by both platforms are presented. Differentially induced genes are colored to highlight genes uniquely identified using microarrays (open red down triangles) or RNA-Seq (open blue up triangles) and found common between the two methods (purple crosses). (**B**) Three-way Venn comparing differentially expressed genes identified from GENE-counter's assessment of RNA-Seq data and analysis of microarrays. Only genes measurable using both methods were included in the comparison.

The log_2_-fold change of expression for the induced genes identified from the two methods was well correlated (Fig. 4A). As previously noted, stronger correlations were noted for genes with higher levels of expression [50]. Importantly, analysis of microarrays gave strong support for the genes found by GENE-counter and measurable using microarrays, 174 of 254 or 68% of the induced genes, were common to both expression platforms (Fig. 4B). Additionally, of 22 randomly selected induced genes, 20 were confirmed as differentially induced using qRT-PCR (≥2-fold relative expression; data not shown). We also compared results from an independent microarray study most similar to ours, infection of Arabidopsis with a Δ*hrpA* T3SS mutant of *Pto*DC3000 at 6 hpi [46]. We used the same methods to reanalyze these data and arrived at 414 differentially induced genes, which when compared, supported 58% and 57% of the differentially induced genes identified using GENE-counter and analysis of our microarrays, respectively. Between the two microarray studies, 78% of the differentially induced genes identified using GENE-counter, and measurable by both methods, were supported. Collectively, our analyses suggested the majority of the genes identified using GENE-counter are *bona fide* differentially induced genes.

### Comparison to Cufflinks

We compared the performance of GENE-counter to Cufflinks version 1.0.2. For alignments, Cufflinks uses Bowtie with a genome reference sequence and TopHat with an optional transcriptome reference annotation to identify splice junctions and guide inference of transcript isoforms, respectively [16]. In contrast, with GENE-counter, an end user can specify genome, transcriptome, or both reference sequences for alignments. A total of 27,968,144 reads were found to be unambiguous and usable based on alignments by GENE-counter. Cufflinks, when given this set of reads, aligned 26,873,027 to the genome and 735,520 to splice junctions. This compared favorably to GENE-counter, which aligned 26,976,496 to the genome and 991,648 to the transcriptome reference sequence. There were some rare and notable differences but they are not expected to be of much consequence; for example, 16,784 reads used by TopHat to infer splice junctions were aligned to the genome reference sequence by CASHX. As expected with the similarities in alignments, there were high correlations in mean gene expression levels for both treatments (Fig. S1).

Despite the congruence of results up to this step of the two pipelines, only ∼24% of the 260 differentially induced and significant genes identified by Cufflinks overlapped with the original NBPSeq set of 308 genes (Table S4). Only ∼10% of the genes unique to Cufflinks were identified in a minimum of at least one microarray study, with the majority of those found in only one [42]–[49]. In contrast, ∼86% of genes unique to GENE-counter were often identified across several microarray studies (data not shown). Additional attempts that included increasing the ‘minimum alignment count’ of Cufflinks to filter out low expressing genes, using all reads in Cufflinks, using Bowtie for alignments to skip isoform predictions by Cufflinks, and comparing results to GENE-counter using an exon only reference database for alignments, resulted in no substantial increases in overlap of gene lists (data not shown). Therefore, our comparisons show that, with the settings, databases, and data used, the final outputs of GENE-counter and Cufflinks were dissimilar with no more than 30% overlap.

Results from independent statistics packages and expression platforms were largely in agreement with results from GENE-counter but the same cannot be said for Cufflinks. The different strategies for measuring isoform versus gene expression could partially explain the discrepancy in results. A study suggested that Cufflinks (ver. 1.0.0), but not methods like GENE-counter, could reliably identify differentially expressed genes when simulated total gene counts were held constant and expression was switched *in silico* from all isoforms in one group to exclusively a single isoform in another group [51]. This is, however, a unique and extreme case and unlikely generalizable to all genes that differed in the comparisons.

The pilot RNA-Seq dataset could also have contributed to the observed differences as statistical analysis of RNA-Seq data has suggested that technical variability can be substantial and is further exacerbated with lower depth of sequencing [52]. We have used GENE-counter to analyze other RNA-Seq datasets and in these few cases, greater depth of sequencing did not appear to improve results. Particularly informative were two independent rRNA-depleted RNA-Seq experiments of *in vitro* grown bacteria. The depth of sequencing amply exceeded the depth achieved with the Arabidopsis dataset and furthermore, analyses were not complicated by the presence of alternatively spliced isoforms. Nevertheless, in one experiment the overlap in differentially expressed genes identified using GENE-counter and Cufflinks was still less than 30% (J. Dangl, and C. Jones; personal communication). In the other, the number of genes identified using Cufflinks was slightly more than 20% the number found using GENE-counter (J. Kimbrel and J. Chang, unpublished).

There are differences in the statistical methods used by the two pipelines. Uncertainties in read assignments are addressed by Cufflinks using maximum likelihood estimates. This approach has the potential to impact conclusions on differential gene expression [51]. Secondly, Cufflinks uses a different statistical test than GENE-counter, but this is very likely minor. It is also unclear to us whether Cufflinks uses an important statistical power saving feature that is used by all three statistics packages configured in GENE-counter. We are reluctant in speculating whether these explain the differences in results as Cufflinks experienced substantial and multiple recent changes. We encourage end users to consider and test both pipelines to identify the method most suitable for their purposes.

One important consideration is that GENE-counter does not infer transcript isoforms or directly examine their differential expression. This, however, does not preclude the use of GENE-counter for studying differential expression of transcript isoforms. End users can select genome, transcriptome, or both types of reference databases for alignments. The transcriptome databases for many model organisms are continuously updated to include newly discovered transcript isoforms and when combined with the rapid advances in next-gen technology, may contribute to more accurate alignments of RNA-Seq reads to resolve transcript isoforms and homologous genes. Many software programs for *de novo* assembly of transcripts as well as empirical identification of splice junctions and inference of splice variants from RNA-Seq reads are available [16], [53]–[55]. These programs could be used to first develop a transcript isoform database with empirically supported sequences. This database could then be used by GENE-counter to identify differentially expressed transcript isoforms.

In summary, GENE-counter is a pipeline for analyzing RNA-Seq data for differential gene expression. Its strengths include ease of use, modularity, appropriateness of statistical tests, and systematic storage of data. Additionally, GENE-counter is well suited for studying gene expression changes of prokaryotes as well as non-model organisms with only a transcriptome reference sequence first inferred directly from the RNA-Seq data using other software programs. GENE-counter and its user's manuals can be downloaded from our website at: http://changlab.cgrb.oregonstate.edu/. GENE-counter is also available for download from sourceforge.net. Note added in Proof: Cufflinks version 1.1.0 was released on 9/8/2011 that includes a check for sufficiency in depth of sequencing and fixes a bug in the calculation of the parameters for the NB distribution (http://cufflinks.cbcb.umd.edu/).

## Supporting Information

Figure S1 **Comparison of the log of the mean gene expression values determined by GENE-counter and Cufflinks.**
(PDF)

Table S1 **Differentially expressed genes identified using GENE-counter running NBPSeq.**
(XLS)

Table S2 **Enriched GO terms for the original set of differentially induced genes.**
(XLS)

Table S3 **Differentially induced genes from analysis of microarrays.**
(XLS)

Table S4 **Differentially expressed genes identified using Cufflinks.**
(XLS)
