# Supplementary material for: GENE-Counter: A Computational Pipeline for the Analysis of RNA-Seq Data for Gene Expression Differences
Source: PLoS One. 2011 Oct 6;6(10):e25279. doi: 10.1371/journal.pone.0025279 (PMC3188579; doi:10.1371/journal.pone.0025279)
Supplement: Figure S1 — Comparison of the log of the mean gene expression values determined by GENE-counter and Cufflinks. (PDF) [file pone.0025279.s001.pdf]

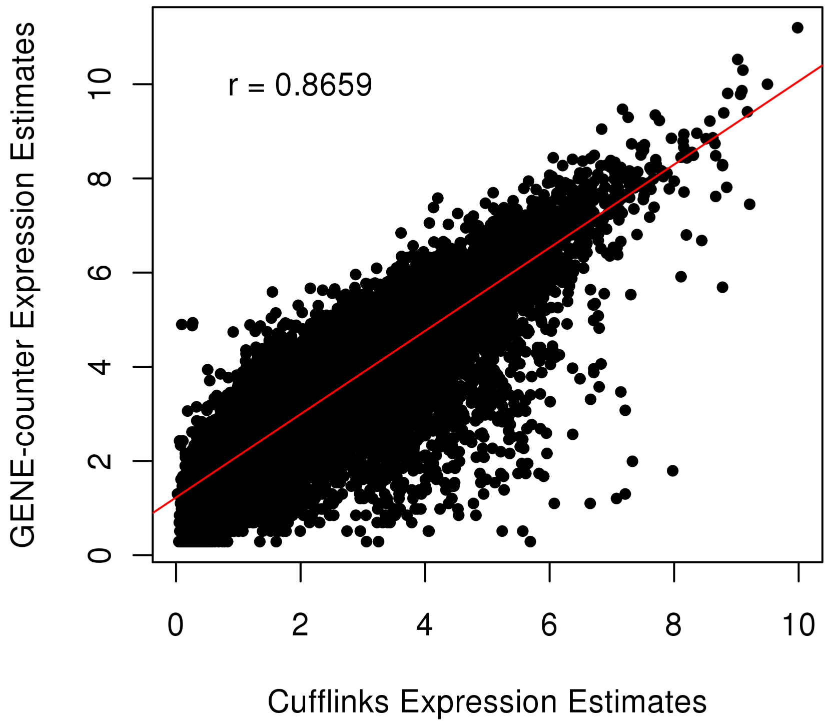

Fig. S1: Comparison of the log of the mean gene expression values determined by GENE-counter and Cufflinks. The average of the log transformed normalized expression values derived for genes from Cufflinks (x-axis) and GENE-counter (y-axis) were plotted against each (only Δ*hrcC* shown; mock was similar (r = 0.86)). Only genes tested in both packages with an estimated expression > 0 by either package (Δ*hrcC* = 19168 and mock = 19047) were included. In cases where a gene had more than one expressed isoform, the isoform with the highest expression level was used. All log-transformed values were treated as described in the main text. Pearson's correlation coefficients and the scatter plot were calculated and created as described in the main text.
